# Differential pharmacological and sex-specific effects of antimuscarinic agents at the hypoglossal motor nucleus in vivo in rats

**DOI:** 10.1038/s41598-022-19233-1

**Published:** 2022-09-01

**Authors:** Sepehr Niakani, Hattie Liu, Wen-Ying Liu, Richard L. Horner

**Affiliations:** 1 Department of Physiology, University of Toronto, Toronto, Canada; 2 Department of Medicine, University of Toronto, 3206 Medical Sciences Building, 1 King’s College Circle, Toronto, ON M5S 1A8 Canada

**Keywords:** Neurophysiology, Respiration

## Abstract

Successful cholinergic-noradrenergic pharmacotherapy for obstructive sleep apnea (OSA) is thought to be due to effects at the hypoglossal motor nucleus (HMN). Clinical efficacy varies with muscarinic-receptor (MR) subtype affinities. We hypothesized that oxybutynin (cholinergic agent in successful OSA pharmacotherapy) is an effective MR antagonist at the HMN and characterized its efficacy with other antagonists. We recorded tongue muscle activity of isoflurane anesthetized rats (121 males and 60 females, 7–13 per group across 13 protocols) in response to HMN microperfusion with MR antagonists with and without: (i) eserine-induced increased endogenous acetylcholine at the HMN and (ii) muscarine. Eserine-induced increased acetylcholine decreased tongue motor activity (p < 0.001) with lesser cholinergic suppression in females versus males (p = 0.017). Motor suppression was significantly attenuated by the MR antagonists atropine, oxybutynin, and omadacycline (MR2 antagonist), each p < 0.001, with similar residual activity between agents (p ≥ 0.089) suggesting similar efficacy at the HMN. Sex differences remained with atropine and oxybutynin (p < 0.001 to 0.05) but not omadacycline (p = 0.722). Muscarine at the HMN also decreased motor activity (p < 0.001) but this was not sex-specific (p = 0.849). These findings have translational relevance to antimuscarinic agents in OSA pharmacotherapy and understanding potential sex differences in HMN suppression with increased endogenous acetylcholine related to sparing nicotinic excitation.

## Introduction

Obstructive sleep apnea (OSA) is a common and widespread disorder with serious clinical, social, and economic problems^1,2^. OSA remains undertreated due to poor compliance with continuous positive airway pressure, the leading therapy^3,4^.

Findings in animal studies identify an endogenous noradrenergic drive that tonically excites the hypoglossal motor nucleus (HMN) in wakefulness with this excitation withdrawn in sleep^5–9^, and a muscarinic receptor inhibitory mechanism that operates especially in rapid eye movement sleep^10^. Such findings identify the chief mechanisms controlling motor output to the pharyngeal muscle in sleep that is central to OSA in humans, and led to selection of cholinergic-noradrenergic drug combinations and reproducible improvements in OSA severity by pharmacotherapy^11–15^.

The common assumption or underlying rationale from those clinical studies^11–14,16,17^ and accompanying editorials^18–20^, is that the agents are likely effective via their actions at the HMN. This assumption needs to be tested to pinpoint mechanism of action and stimulate identification of potentially more effective strategies including the targeting of specific pharmacological profiles: e.g., clinical efficacy varies with muscarinic receptor subtype affinities of the agents tested^11–14,16,17^.

In the present study, respiratory muscle activities are recorded during microperfusion of the HMN in rats with oxybutynin (part of effective OSA pharmacotherapy^11,15,16,21,22^), omadacycline (recently FDA-approved agent and selective muscarinic receptor subtype 2 (M2) antagonist^23,24^), and atropine and/or scopolamine, broad spectrum muscarinic receptor antagonists with the latter also a component of effective OSA pharmacotherapy^13,19^. Acetylcholinesterase inhibition at the HMN to increase endogenous acetylcholine leads to muscarinic receptor-mediated inhibition of motor activity that dominates nicotinic receptor-mediated excitation^25–27^. Accordingly, this study also furthered an established protocol to use eserine-induced increased endogenous acetylcholine at the HMN to modulate muscarinic and nicotinic receptors, and muscarine at the HMN to target only muscarinic receptors^25^. Isoflurane was chosen as the inhalational anesthetic to enable characterization of tongue motor responses to controlled time-dependent sequential delivery of selected interventions into the HMN in vivo across 13 related protocols in male and female rats^25,28^.

The findings of differential pharmacological and sex-specific effects of select antimuscarinic agents at the HMN in vivo have translational relevance to antimuscarinic OSA pharmacotherapy, and to understanding sex differences in HMN suppression with increased endogenous acetylcholine that relate to the sparing of accompanying nicotinic receptor-mediated excitation.

## Results

### Sites of microdialysis perfusion

**Figure 1:**
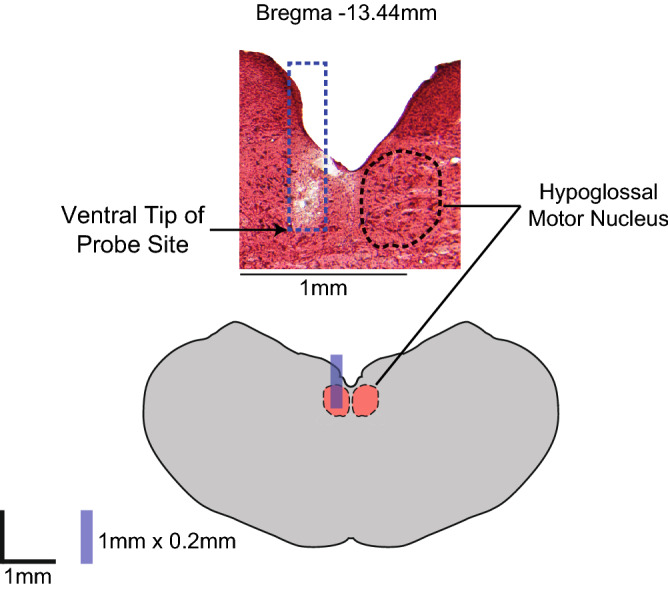
Example of site of microdialysis at the hypoglossal motor nucleus (HMN). The ventral tip of the microdialysis probe identified from the tissue disruption left by the probe is indicated by the arrow. The reconstruction of the probe position and the contralateral HMN outlined by a dotted line are shown (top figure). A schematic representation of the coronal section of medulla 13.44 mm posterior to bregma is also shown with the reconstructed probe position drawn to scale (bottom figure).

Figure 1 shows an example site of microdialysis at the HMN from one experiment and the reconstruction of the location on a corresponding coronal section of medulla. The distributions of probe sites for each experiment in each protocol are included with the respective group data in subsequent figures (Figs. 3, 4, 5, 6, 7, 10, 11, 12, 13, 14). The sites of microdialysis were within or immediately adjacent to the HMN in all experiments across all protocols.

### Study 1: Cholinergic modulation of the HMN by eserine and effect of select muscarinic receptor antagonists

#### Protocol 1.1: Tongue motor inhibition by eserine

**Figure 2:**
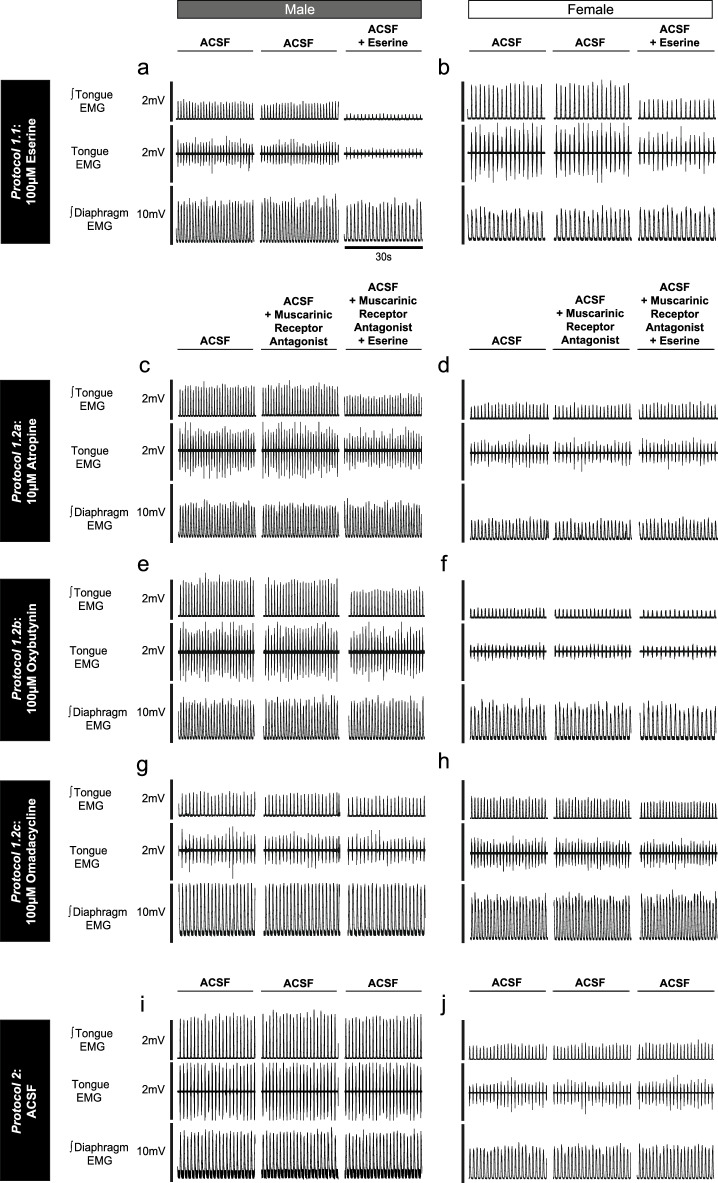
Example traces showing tongue motor inhibition by eserine at the HMN (*Protocol 1.1*), the capacity for its blockade by muscarinic receptor antagonists (*Protocol 1.2a-c*), and respiratory muscle activities over sequential ACSF (i.e., sham) time control interventions (*Protocol 2*). The sets of traces (**a**–**j**) show the raw tongue EMG activity, and the moving time averages (∫) of tongue and diaphragm EMG signals for males (left column) and females (right column). The indicated variables and their scales on the ordinate axes for the left panels (males) are the same as for the adjacent panels on the right (females). The 30-s sample traces represent the last minute of each intervention, with this last-minute period used for data analyses.

**Figure 3:**
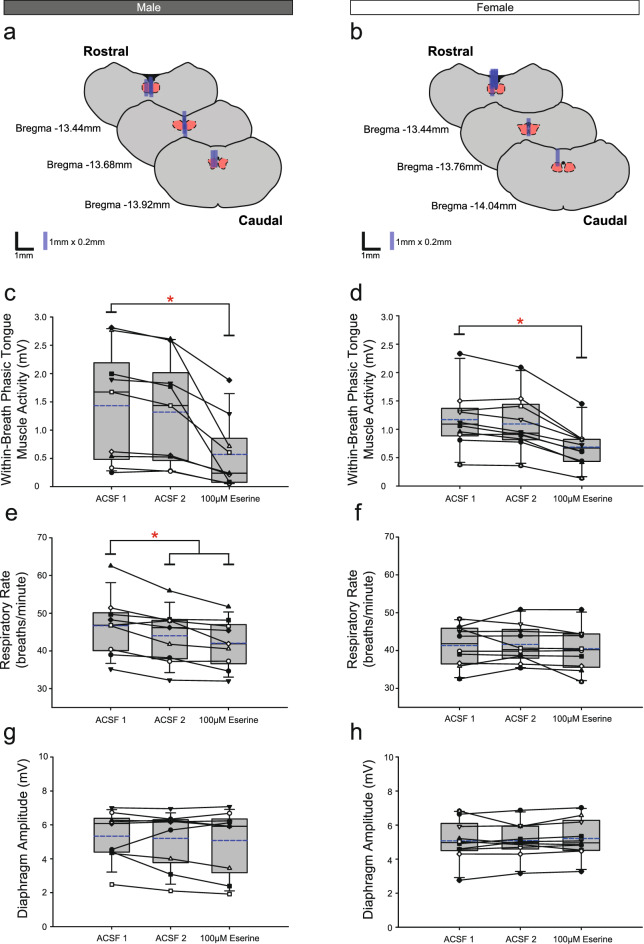
Group data from *Protocol 1.1*: Microperfusion of 100 μM eserine into the HMN (left column: male, n = 9; right column: female, n = 10). Schematic representations of coronal sections of medulla illustrating the distribution of probe sites across all experiments (**a**,**b**). Drawn to scale, the blue rectangles represent reconstruction of the sites of the microdialysis probes; overlap obscures some of the individual probe sites. Box and whisker plots show individual and group data (median [dashed line], mean [solid line] and 25th and 75th percentiles) for within-breath phasic tongue muscle activity (**c**,**d**), respiratory rate (**e**,**f**), and diaphragm amplitude (**g**,**h**). Each animal is indicated by a different symbol. *, indicates p < 0.05 compared to baseline ACSF controls.

Representative traces of the decrease in tongue motor activity in response to microperfusion of eserine into the HMN are shown in Fig. 2a,b for male and female rats respectively. Group data are shown in Fig. 3. Figure 3a,b illustrate the distribution of probe sites from all experiments in *Protocol 1.1*.

#### Within-breath phasic tongue muscle activity

There was a significant effect of the interventions on within-breath phasic tongue muscle activity in males (F_2,16_ = 13.05, p < 0.001, one-way ANOVA-RM) and females (F_2,18_ = 53.04, p < 0.001), with significant decreases with eserine compared to baseline artificial cerebrospinal fluid (ACSF) in both groups (both males and females: p < 0.001, post hoc Dunnett’s tests).

#### Other respiratory parameters

There was no effect on respiratory rate over the intervention periods in female rats (F_2,18_ = 1.01, p = 0.383, one-way ANOVA-RM) but a decrease occurred in male rats (F_2,16_ = 12.93, p < 0.001) during the second ACSF period (p = 0.019, post hoc Dunnett’s test). There were no significant effects on diaphragm amplitude observed in either males or females over the experiments (F_2,16_ = 0.60, p = 0.563 and F_2,18_ = 0.97, p = 0.398 respectively).

#### Protocol 1.2: Modulation of eserine-induced cholinergic hypoglossal motor inhibition by select antimuscarinic agents at the HMN

**Figure 4:**
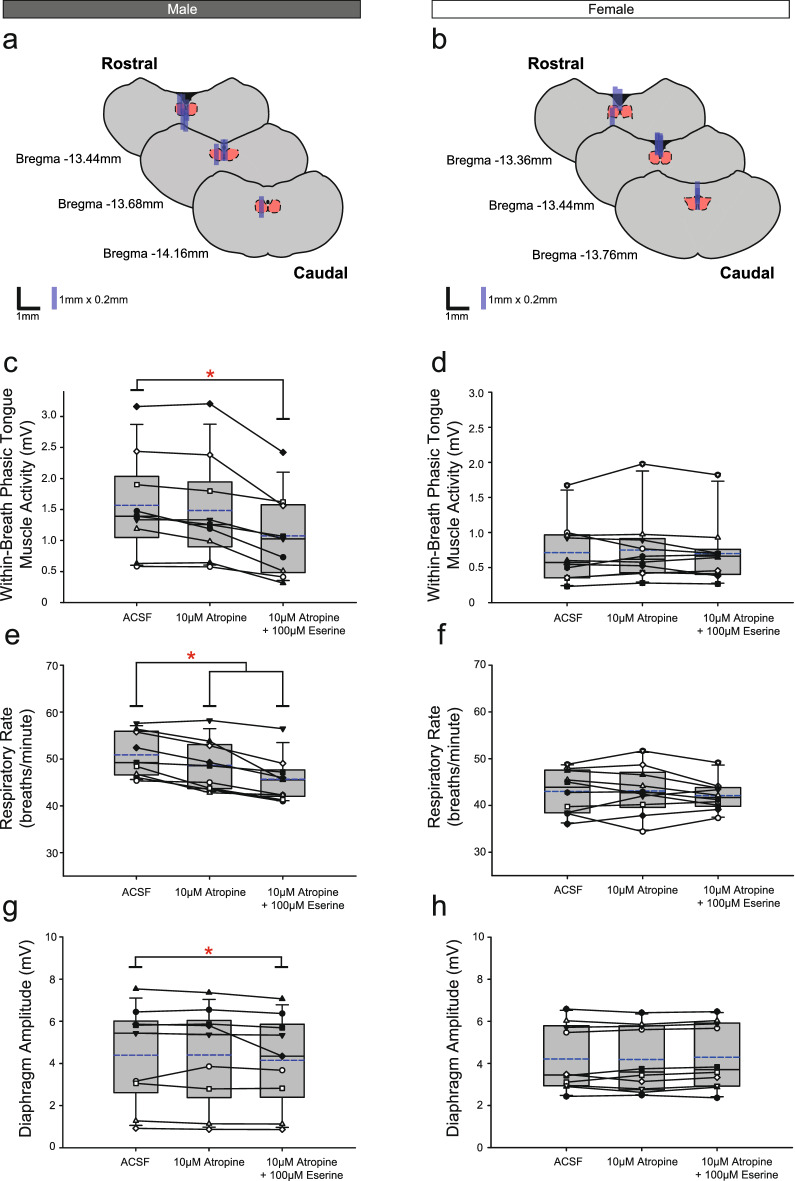
Group data from *Protocol 1.2a*: Microperfusion of 10 μM atropine into the HMN with and without co-application of eserine (left column: male, n = 9; right column: female, n = 10). Schematic representations of coronal sections of medulla illustrating the distribution of probe sites across all experiments (**a**,**b**). Drawn to scale, the blue rectangles represent reconstruction of the sites of the microdialysis probes; overlap obscures some of the individual probe sites. Box and whisker plots show individual and group data (median [dashed line], mean [solid line] and 25th and 75th percentiles) for within-breath phasic tongue muscle activity (**c**,**d**), respiratory rate (**e**,**f**), and diaphragm amplitude (**g**,**h**). Each animal is indicated by a different symbol. *, indicates p < 0.05 compared to baseline ACSF controls.

**Figure 5:**
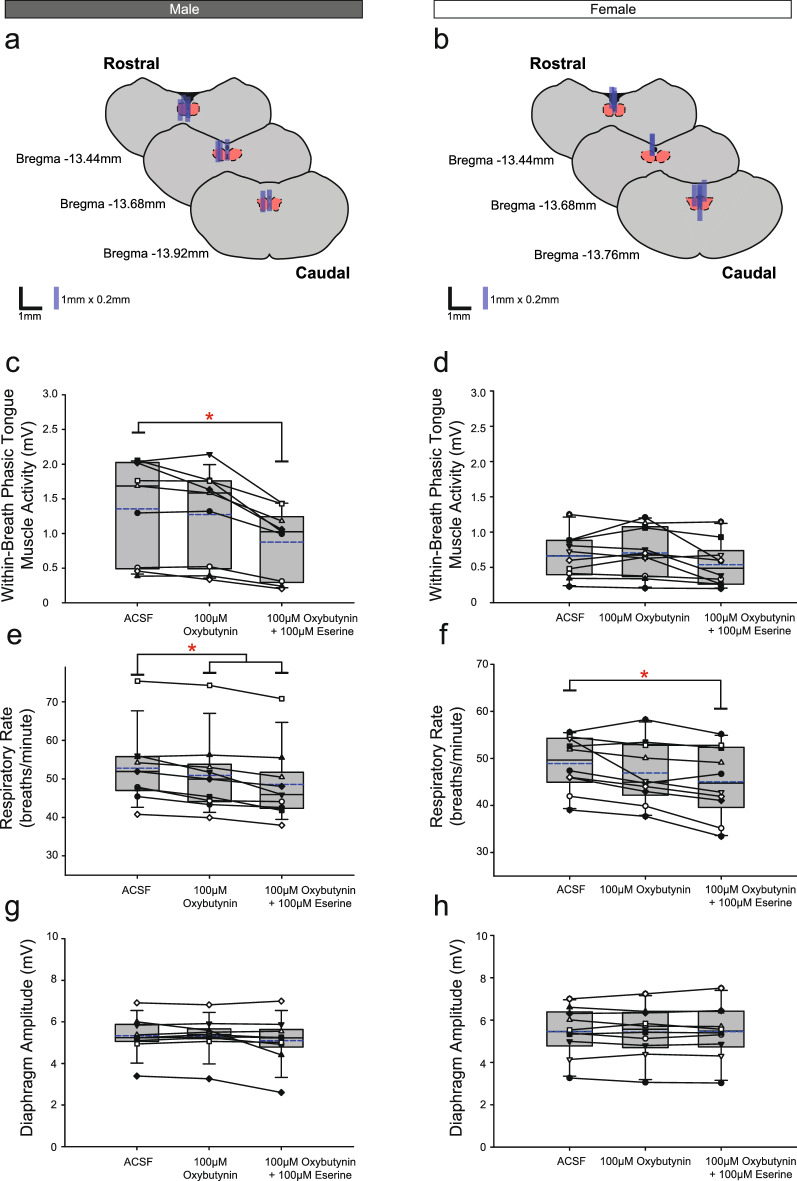
Group data from *Protocol 1.2b*: Microperfusion of 100 μM oxybutynin into the HMN with and without co-application of eserine (left column: male, n = 9; right column: female, n = 10). Schematic representations of coronal sections of medulla illustrating the distribution of probe sites across all experiments (**a**,**b**). Drawn to scale, the blue rectangles represent reconstruction of the sites of the microdialysis probes; overlap obscures some of the individual probe sites. Box and whisker plots show individual and group data (median [dashed line], mean [solid line] and 25th and 75th percentiles) for within-breath phasic tongue muscle activity (**c**,**d**), respiratory rate (**e**,**f**), and diaphragm amplitude (**g**,**h**). Each animal is indicated by a different symbol. *, indicates p < 0.05 compared to baseline ACSF controls.

**Figure 6:**
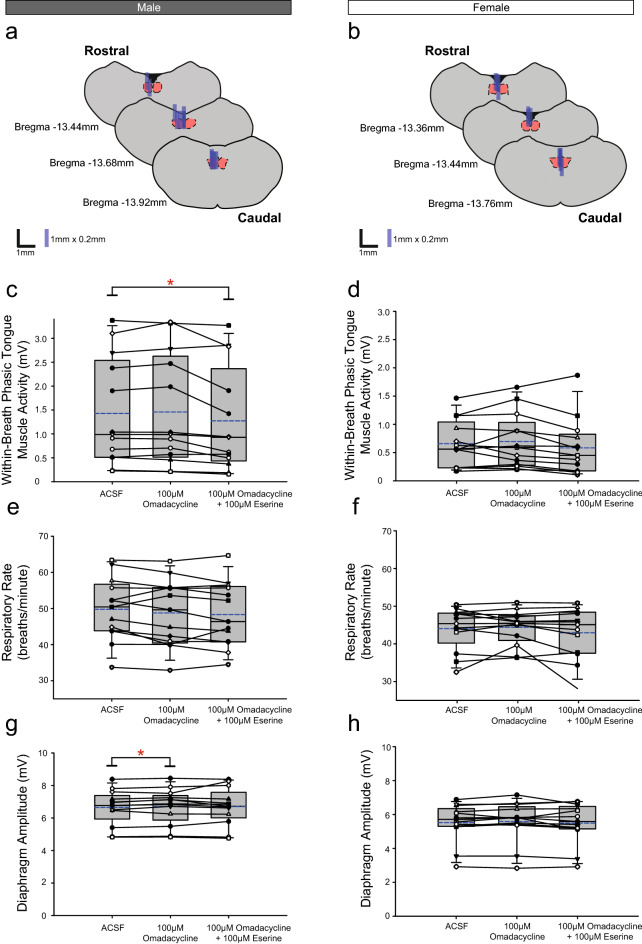
Group data from *Protocol 1.2c*: Microperfusion of 100 μM omadacycline into the HMN with and without co-application of eserine (left column: male, n = 13; right column: female, n = 13). Schematic representations of coronal sections of medulla illustrating the distribution of probe sites across all experiments (**a**,**b**). Drawn to scale, the blue rectangles represent reconstruction of the sites of the microdialysis probes; overlap obscures some of the individual probe sites. Box and whisker plots show individual and group data (median [dashed line], mean [solid line] and 25th and 75th percentiles) for within-breath phasic tongue muscle activity (**c**,**d**), respiratory rate (**e**,**f**), and diaphragm amplitude (**g**,**h**). Each animal is indicated by a different symbol. *, indicates p < 0.05 compared to baseline ACSF controls.

**Figure 7:**
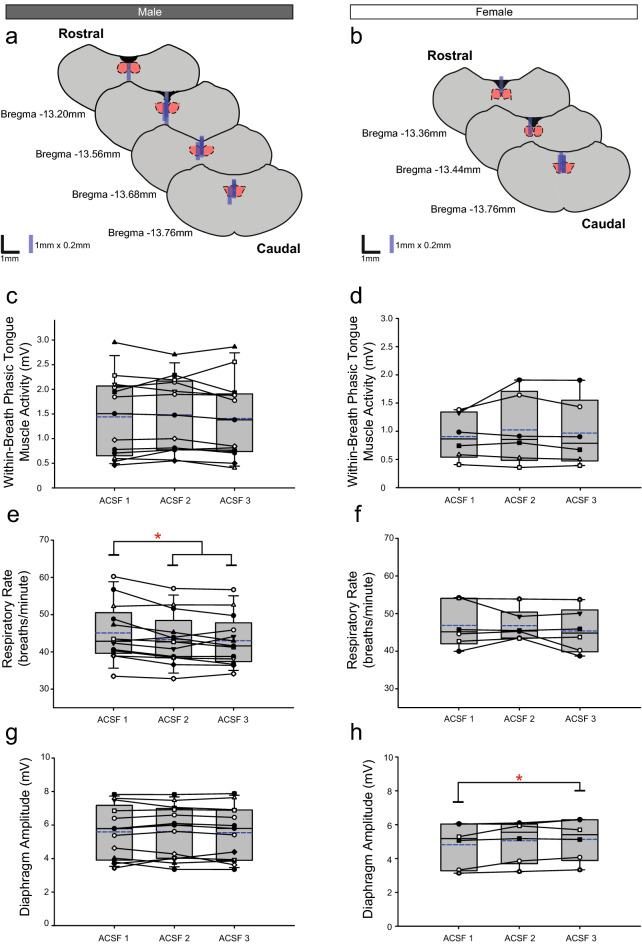
Group data from *Protocol 2*: Effects of sequential ACSF (i.e., sham) time-control interventions at the HMN (left column: male, n = 13; right column: female, n = 6). Schematic representations of coronal sections of medulla illustrating the distribution of probe sites across all experiments (**a**,**b**). Drawn to scale, the blue rectangles represent reconstruction of the sites of the microdialysis probes; overlap obscures some of the individual probe sites. Box and whisker plots show individual and group data (median [dashed line], mean [solid line] and 25th and 75th percentiles) for within-breath phasic tongue muscle activity (**c**,**d**), respiratory rate (**e**,**f**), and diaphragm amplitude (**g**,**h**). Each animal is indicated by a different symbol. *, indicates p < 0.05 compared to baseline ACSF controls.

Figure 2 shows representative example responses across the interventions in each of *Protocols 1.2a-c* in both male and female rats. The respective group data and sites of microdialysis are shown in Figs. 4, 5 and 6, with time control data shown in Fig. 7.

#### Within-breath phasic tongue muscle activity

Analysis of the group data for *Protocols 1.2a* (atropine, Fig. 4c,d), *1.2b* (oxybutynin, Fig. 5c,d) and *1.2c* (omadacycline, Fig. 6c,d) identified statistically significant effects of the interventions on within-breath phasic tongue muscle activity in male rats in each protocol (F_2,16_ = 26.50, p < 0.001, F_2,16_ = 19.27, p < 0.001, and F_2,24_ = 8.44, P = 0.002, respectively, one-way ANOVA-RM in each of Figs. 4, 5 and 6 respectively), with statistically significant decreases compared to the baseline ACSF condition occurring in the presence of co-applications of atropine, oxybutynin or omadacycline with eserine (p < 0.001, p < 0.001 and p = 0.007, respectively, post hoc Dunnett’s tests).

**Figure 8:**
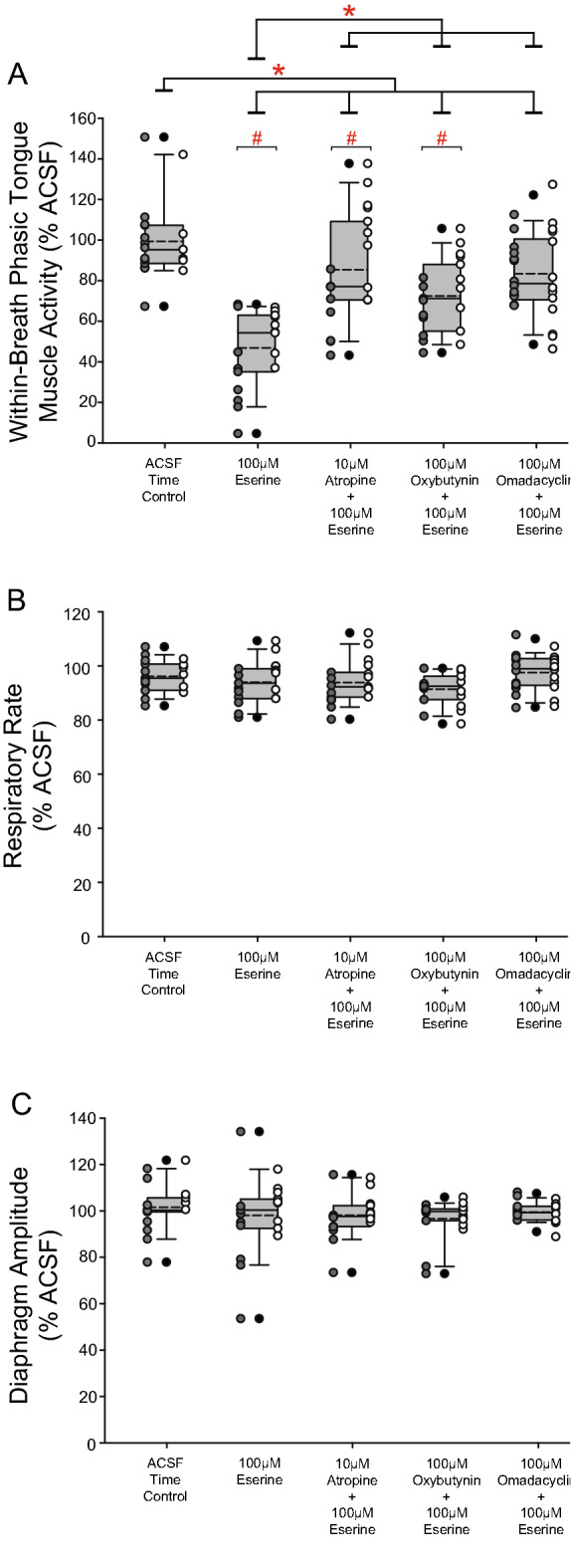
Relative efficacy of the antimuscarinic agents at the HMN in Study 1 in reducing eserine-induced tongue motor suppression and ACSF time controls. Individual data (males, gray round symbols on left of each bar; females, white symbols on right of each bar) and group data (population of males and females combined, grey bar) showing respiratory motor responses across protocols. Data are normalized to the baseline ACSF control period in each individual. Box and whisker plots show individual and group data comprising the plot (median (dashed line), mean (solid line), 25th and 75th percentiles) for within-breath phasic tongue muscle activity (**a**), respiratory rate (**b**), and diaphragm amplitude (**c**). * and # indicate p ≤ 0.05.

These data show that a level hypoglossal motor suppression remained after application of the muscarinic receptor antagonists in the presence of increased endogenous acetylcholine. Of note, comparisons of the relative magnitude of eserine-induced suppression of tongue motor activity with and without co-application of these antagonists with different muscarinic receptor subtype-preferring profiles is identified in a later summary analysis (Fig. 8). Overall, tongue motor activity in the presence of eserine-increased acetylcholine at the HMN was significantly lower (i.e., greater suppression) compared to when each of atropine, oxybutynin and omadacycline were co-applied at the HMN in male and female rats (Fig. 8) indicating a significant effect of the antagonists (see below for detailed analyses and comparisons within and between protocols). Also of note, the resulting level of tongue motor activity with eserine-induced increased endogenous acetylcholine is the net result of muscarinic receptor-mediated inhibition of hypoglossal motor activity that dominates nicotinic receptor-mediated excitation^25–27^. Overall, the net activity after co-application of the different muscarinic-receptor antagonists with eserine is the result of the prevailing balance of such excitatory and inhibitory effects.

Significant effects of the interventions on within-breath phasic tongue muscle activity were also identified in female rats for *Protocol 1.2b* (oxybutynin, Fig. 5, F_2,18_ = 5.06, p = 0.018, one-way ANOVA-RM) but not for *Protocols 1.2a and 1.2c* (atropine and omadacycline, Figs. 4 and 6, F_2,18_ = 0.79, p = 0.471, and F_2,24_ = 3.07, p = 0.065, respectively).

In each of *Protocols 1.2a-c*, there was no effect of microperfusion of the muscarinic-receptor antagonist *alone* into the HMN on within-breath phasic tongue muscle activity of male (all p ≥ 0.440, post hoc Dunnett’s test) or female rats (p = 0.656, post hoc Dunnett’s test).

#### Other respiratory parameters

Comparison of Figs. 4, 5 and 6 (panels e and f) for each of the *Protocols 1.2a-c* show a general trend of a decline in respiratory rate over the course of the experiments for both male rats (statistically significant decreases are indicated by the symbol ‘*’, each p ≤ 0.026, post hoc Dunnett’s test after significant one-way ANOVA- RM, all F ≥ 19.21, all p < 0.001) and female rats (p < 0.001, post hoc Dunnett’s test after significant one-way ANOVA-RM, F ≥ 9.07, p = 0.002). A small but statistically significant effect on diaphragm amplitude was observed in male rats in *Protocols 1.2a* and *1.2c* (Figs. 4g and 6g, p = 0.013 and p = 0.049, respectively, post hoc Dunnett’s test one-way ANOVA-RM on Ranks) but not in *Protocol 1.2b* (Fig. 5g, p = 0.328, post hoc Dunnett’s test one-way ANOVA-RM on Ranks). There were no significant effects on diaphragm amplitude in female rats in *Protocols 1.2a-c* (all F ≤ 1.79, all p ≥ 0.196, one-way ANOVA-RM).

As noted below, these effects on respiratory rate occurring during the experiments also similarly occur in the ACSF time controls (Fig. 7) and as such appear due to the general effects of time and not the drug interventions per se.

### Study 2: Time controls

#### Protocol 2: Time controls with continued microperfusion of ACSF into the HMN

Figure 2 includes representative traces of respiratory EMG activities occurring over sequential microperfusion of ACSF into the HMN (i.e., sham interventions) from *Protocol 2*. Group data are shown in Fig. 7.

#### Within-breath phasic tongue muscle activity

Analysis identified no significant effects of sequential microperfusion of ACSF into the HMN on within-breath tongue muscle activity of male (F_2,24_ = 0.90, p = 0.419, one-way ANOVA-RM, Fig. 7a) or female rats (F_2,10_ = 0.90, p = 0.436, Fig. 7d).

#### Other respiratory parameters

Consistent with previous protocols, compared to the baseline ACSF controls there was a statistically significant decline in respiratory rate observed over the course of the experiments for male rats (F_2,24_ = 6.32, p = 0.006, one-way ANOVA-RM, each p ≤ 0.017, post hoc Dunnett’s test, significant differences indicated by the symbol ‘*’ in Fig. 7e) but not for female rats (F_2,10_ = 1.20, p = 0.340, one-way ANOVA-RM). A significant change (small increase) in diaphragm amplitude was identified in one of the protocols in female rats (Fig. 7h, p = 0.016, post hoc Dunnett’s test after significant one-way ANOVA-RM, F_2,10_ = 5.68, p = 0.023,) but not in the males (F_2,24_ = 0.25, p = 0.781, one-way ANOVA-RM).

### Relative efficacy of the antimuscarinic agents at the HMN in reducing eserine-induced tongue motor suppression

#### Within-breath phasic tongue muscle activity

Figure 8 shows group data comparing within-breath phasic tongue muscle responses across all protocols with eserine-induced increased endogenous acetylcholine and the respective time-controls. There was a significant effect of the interventions across the different protocols on tongue motor activity (F_4,92_ = 21.22, p < 0.001, two-way ANOVA), and effects that were sex specific (F_4,92_ = 3.54, p = 0.010, two-way ANOVA), see symbols ‘*’ and ‘#’ for each of these intervention and sex effects respectively.

#### Relative effects of the different antimuscarinic agents

Within-breath phasic tongue muscle activity in the presence of eserine-increased acetylcholine at the HMN was significantly lower (i.e., greater suppression) compared to when each of atropine, oxybutynin and omadacycline were co-applied at the HMN in male and female rats (all p < 0.001, post hoc Holm–Sidak tests, see symbols ‘*’ in Fig. 8a with eserine and then with atropine, oxybutynin and omadacycline). These data show that there was significant antagonism of the eserine-induced suppression of tongue motor activity by the respective muscarinic receptor antagonists at the HMN. Also notably, there were no statistically significant differences in the resulting levels of tongue motor activity between atropine and oxybutynin (p = 0.089, post hoc Holm–Sidak test), atropine and omadacycline (p = 0.956, post hoc Holm–Sidak test), and oxybutynin and omadacycline (p = 0.104, post hoc Holm–Sidak test).

Overall, these data showed that there was comparable efficacy of these interventions at the HMN in reducing tongue motor suppression in the presence of increased endogenous acetylcholine at the HMN.

#### Sex effects

There were differences in the levels of within-breath phasic tongue muscle activity between male and female rats in the protocols with eserine at the HMN (p = 0.017, post hoc Holm–Sidak test), atropine co-applied with eserine (p < 0.001), and oxybutynin co-applied with eserine (p = 0.05, post hoc Holm–Sidak test, and p = 0.037 unpaired t-test), but not for omadacycline co-applied with eserine (p = 0.722) or across the ACSF time controls (p = 0.795)—see the symbols ‘#’ in Fig. 8a. Overall, these data show that (i) tongue muscle activity was significantly higher with eserine at the HMN in females vs. males (i.e., lesser cholinergic-induced motor suppression), and (ii) the resulting tongue motor activity was also higher (less suppressed) in the presence of co-applied atropine and oxybutynin. This sex-specific effect did not occur in the presence of the M2 receptor specific antagonist omadacycline at the HMN, nor did it occur with the ACSF time controls (each p ≥ 0.722, post hoc Holm–Sidak tests).

#### Other respiratory parameters

Any changes in respiratory rate and diaphragm amplitude over the course of the experiments were similar across all protocols (i.e., there were no protocol-dependent differences). There were no effects on respiratory rate or diaphragm amplitude that were dependent on sex (F_4,92_ = 2.17, p = 0.078 and F_4,92_ = 1.23, p = 0.305, respectively) or drug treatment (F_4,92_ = 2.15, p = 0.081 and F_4,92_ = 1.07, p = 0.374, respectively).

### Study 3: Cholinergic modulation of the HMN by muscarine and effect of select muscarinic receptor antagonists

#### Protocol 3.1: Tongue motor inhibition by muscarine

**Figure 9:**
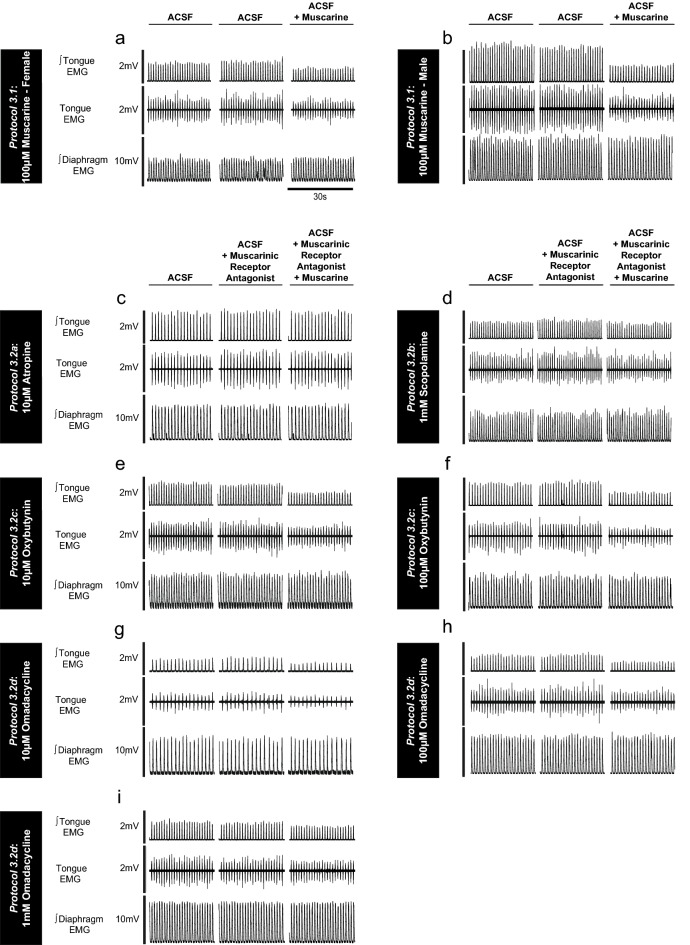
Example traces showing tongue motor inhibition by muscarine at the HMN (*Protocol 3.1*) and the capacity for its blockade by muscarinic receptor antagonists (*Protocol 3.2a-d*). The sets of traces (**a**–**i**) show the raw tongue EMG activity, and the moving time averages (∫) of the tongue and diaphragm EMG signals during microperfusion of muscarine in female and male rats (**a**,**b**) or when co-applied with muscarinic receptor antagonists in male rats (**c**–**i**). The 30-s sample traces represent the last minute of each intervention, with this last-minute period used for data analyses.

**Figure 10:**
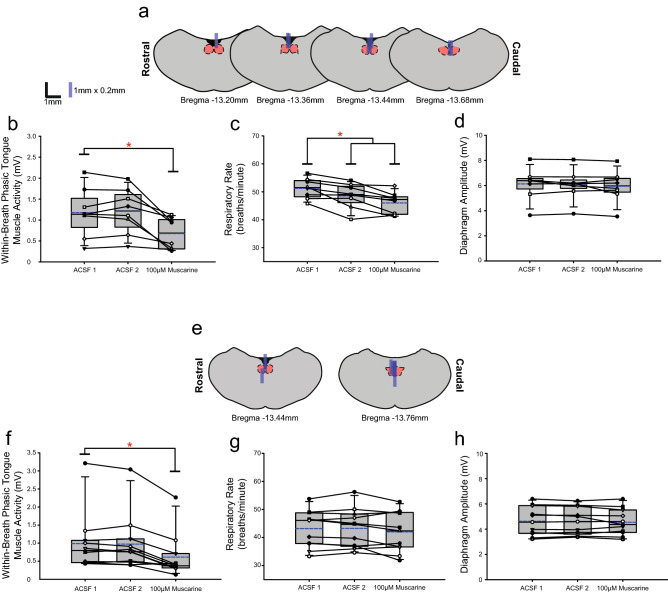
Group data from *Protocol 3.1*: Microperfusion of 100 μM muscarine into the HMN (top panels: male, n = 8; bottom panels: female, n = 11). Schematic representations of coronal sections of medulla illustrating the distribution of probe sites across all experiments (**a**,**e**). Drawn to scale, the blue rectangles represent reconstruction of the sites of the microdialysis probes; overlap obscures some of the individual probe sites. Box and whisker plots show individual and group data (median [dashed line], mean [solid line] and 25th and 75th percentiles) for within-breath phasic tongue muscle activity (**b**,**f**), respiratory rate (**c**,**g**), and diaphragm amplitude (**d**,**h**). Each animal is indicated by a different symbol. *, indicates p < 0.05 compared to baseline ACSF controls.

Representative traces of the decrease in tongue motor activity in response to microperfusion of muscarine into the HMN are shown in Fig. 9a,b for female and male rats respectively. Group data are shown in Fig. 10 and include the distribution of probe sites from all experiments in *Protocol 3.1*.

### Within-breath phasic tongue muscle activity

Figure 10b,f show there was a significant effect of muscarine on within-breath phasic tongue muscle activity in males (F_2,14_ = 12.26, p < 0.001, one-way ANOVA-RM) and females (F_2,20_ = 22.28, p < 0.001), with significant decreases compared to baseline ACSF in both sexes (each p ≤ 0.008).

#### Other respiratory parameters

Figure 10c,g show there was no effect on respiratory rate over the intervention periods in female rats (F_2,20_ = 2.19, p = 0.138, one-way ANOVA-RM) but a decrease occurred in male rats (F_2,14_ = 15.67, p < 0.001) during the second ACSF period (p = 0.023, post hoc Dunnett’s test). Figure 10d,h show there were no significant effects on diaphragm amplitude observed in either males or females over the experiments (F_2,14_ = 1.32, p = 0.229 and F_2,20_ = 0.72, p = 0.500, Fig. 10d,h respectively).

#### Protocol 3.2: Modulation of muscarine-induced hypoglossal motor inhibition by select antimuscarinic agents at the HMN

**Figure 11:**
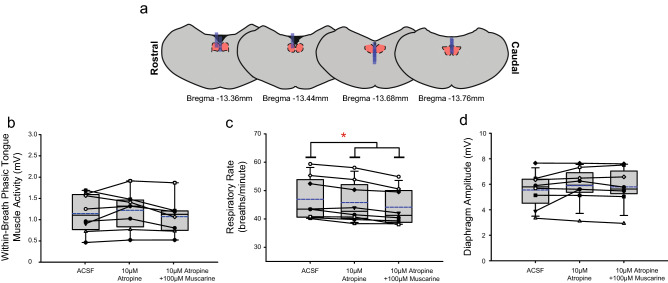
Group data from *Protocol 3.2a*: Microperfusion of 10 μM atropine into the HMN with and without co-application of muscarine (n = 8). Schematic representations of coronal sections of medulla illustrating the distribution of probe sites across all experiments (**a**). Drawn to scale, the blue rectangles represent reconstruction of the sites of the microdialysis probes; overlap obscures some of the individual probe sites. Box and whisker plots show individual and group data (median [dashed line], mean [solid line] and 25th and 75th percentiles) for within-breath phasic tongue muscle activity (**b**), respiratory rate (**c**), and diaphragm amplitude (**d**). Each animal is indicated by a different symbol. *, indicates p < 0.05 compared to baseline ACSF controls.

**Figure 12:**
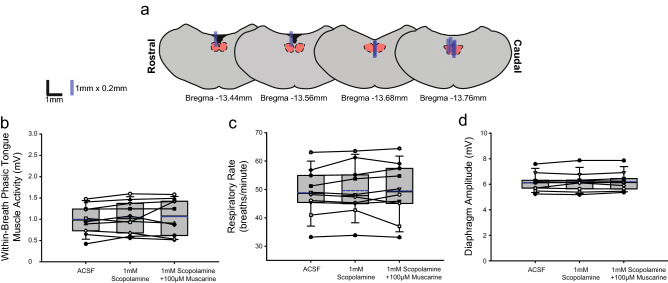
Group data from *Protocol 3.2b*: Microperfusion of 1 mM scopolamine into the HMN with and without co-application of muscarine (n = 10). Schematic representations of coronal sections of medulla illustrating the distribution of probe sites across all experiments (**a**). Drawn to scale, the blue rectangles represent reconstruction of the sites of the microdialysis probes; overlap obscures some of the individual probe sites. Box and whisker plots show individual and group data (median [dashed line], mean [solid line] and 25th and 75th percentiles) for within-breath phasic tongue muscle activity (**b**), respiratory rate (**c**), and diaphragm amplitude (**d**). Each animal is indicated by a different symbol. *, indicates p < 0.05 compared to baseline ACSF controls.

**Figure 13:**
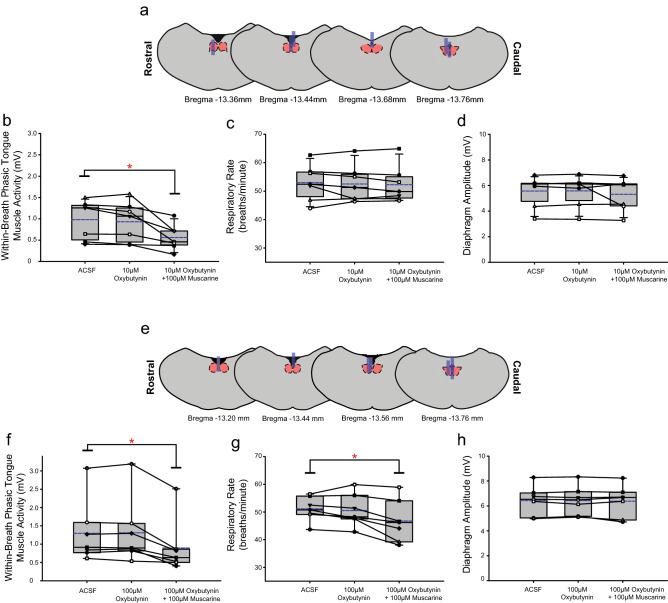
Group data from *Protocol 3.2c*: Microperfusion of oxybutynin into the HMN with and without co-application of muscarine (top panels: 10 µM, n = 7; bottom panels: 100 µM, n = 7). Schematic representations of coronal sections of medulla illustrating the distribution of probe sites across all experiments (**a**,**e**). Drawn to scale, the blue rectangles represent reconstruction of the sites of the microdialysis probes; overlap obscures some of the individual probe sites. Box and whisker plots show individual and group data (median [dashed line], mean [solid line] and 25th and 75th percentiles) for within-breath phasic tongue muscle activity (**b**,**f**), respiratory rate (**c**,**g**), and diaphragm amplitude (**d**,**h**). Each animal is indicated by a different symbol. *, indicates p < 0.05 compared to baseline ACSF controls.

**Figure 14:**
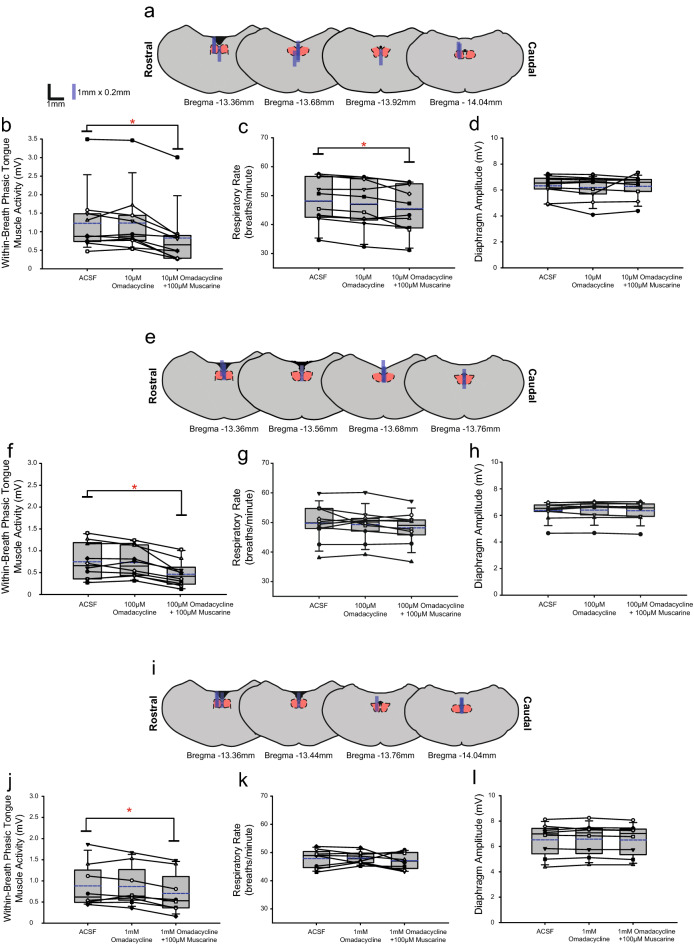
Group data from *Protocol 3.2d*: Microperfusion of omadacycline into the HMN with and without co-application of muscarine (top panels: 10 µM, n = 10; middle panels: 100 µM, n = 10; bottom panels: 1 mM, n = 8). Schematic representations of coronal sections of medulla illustrating the distribution of probe sites across all experiments (**a**,**e**,**i**). Drawn to scale, the blue rectangles represent reconstruction of the sites of the microdialysis probes; overlap obscures some of the individual probe sites. Box and whisker plots show individual and group data (median [dashed line], mean [solid line] and 25th and 75th percentiles) for within-breath phasic tongue muscle activity (**b**,**f**,**j**), respiratory rate (**c**,**g**,**k**), and diaphragm amplitude (**d**,**h**,**l**). Each animal is indicated by a different symbol. *, indicates p < 0.05 compared to baseline ACSF controls.

Figure 9 shows representative example responses across interventions in each of *Protocols 3.2a-d*. Figures 11, 12, 13 and 14 show the respective group data and microdialysis sites.

#### Within-breath phasic tongue muscle activity

Analysis of the group data for *Protocols 3.2a* (atropine, Fig. 11), *3.2b* (scopolamine, Fig. 12), *3.2c* (oxybutynin, Fig. 13) and *3.2d* (omadacycline, Fig. 14) show that in the presence of both atropine and scopolamine at the HMN, there were no changes in within-breath phasic tongue motor activity across the interventions (F_2,14_ = 1.77, p = 0.206, and F_2,18_ = 0.92, p = 0.416, respectively, one-way ANOVA-RM). These results identify that in the presence of these muscarinic receptor antagonists there was no inhibitory effect of muscarine per se on tongue motor activity in contrast to the significant inhibitory effects observed without the antagonists (Figs. 10, 11 and 12), i.e., indicating the capacity for full blockade of muscarinic receptor-mediated inhibition at the HMN.

In contrast, in the presence of oxybutynin (10 and 100 μM, *Protocol 3.2c*, Fig. 13) and omadacycline (10 μM, 100 μM and 1 mM, *Protocol 3.2d*, Fig. 14), statistically significant decreases in within-breath phasic tongue muscle activity occurred in the presence of co-applied muscarine (each F ≥ 11.2 and p ≤ 0.001, one-way ANOVA-RM, all p ≤ 0.002, post hoc Dunnett’s test).

In each of *Protocols 3.2a-d* there was no effect of microperfusion of the muscarinic receptor antagonist alone into the HMN on within-breath phasic tongue muscle activity (all p ≥ 0.824, post hoc Dunnett’s test, Figs. 11, 12, 13 and 14).

#### Other respiratory parameters

Some decreases in respiratory rate were observed in three of the six experiments encompassing *Protocol 3.2a-d* (indicated by the symbol ‘*’ in Figs. 11, 12, 13 and 14, each p ≤ 0.033, post hoc Dunnett’s test after one-way ANOVA-RM indicated significant differences between groups, each F ≥ 7.86 and each p ≤ 0.005). No changes were observed in the other three protocols. As noted above, such effects on respiratory rate also similarly occurred in the ACSF time control experiments (Fig. 7).

There were no effects of the interventions on diaphragm amplitude across *Protocols 3.2a-d* (each F ≤ 1.52 and all p ≥ 0.122, one-way ANOVA-RM, Figs. 11, 12, 13 and 14).

### Relative efficacy of interventions at the HMN to reduce muscarine-induced tongue motor suppression

#### Within-breath phasic tongue muscle activity

**Figure 15:**
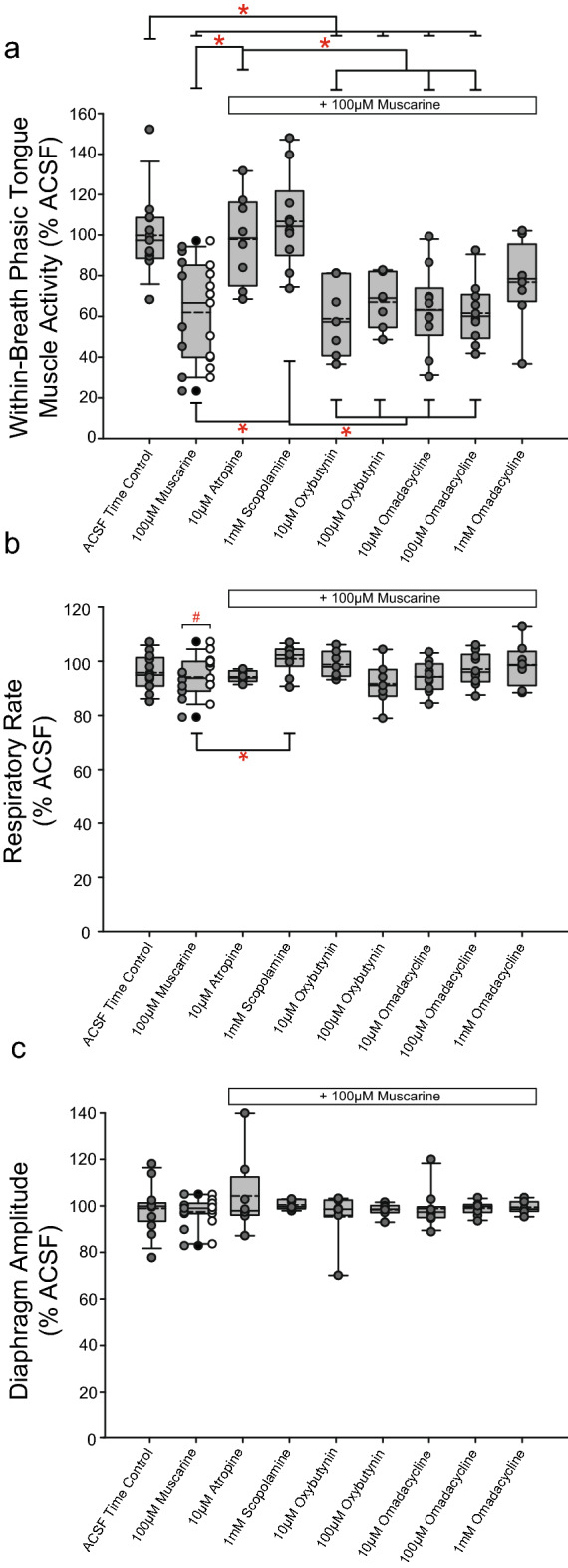
Relative efficacy of the antimuscarinic agents at the HMN in Study 3 in reducing muscarine-induced tongue motor suppression and ACSF time controls. Individual and group data showing respiratory motor responses across protocols. Data are normalized to the baseline ACSF control period in each individual. Box and whisker plots show individual and group data comprising the plot (median [dashed line], mean [solid line] and 25th and 75th percentiles) for within-breath phasic tongue muscle activity (**a**), respiratory rate (**b**), and diaphragm amplitude (**c**). Male and female responses for the protocols with muscarine only are indicated by gray and white circles respectively. *, indicates p < 0.05.

Figure 15 shows group data comparing within-breath phasic tongue muscle responses across all protocols with muscarine-induced inhibition, effects of the antagonists and the respective time-controls. There was a significant effect of the interventions across the different protocols on tongue motor activity (F_8,72_ = 8.17, p < 0.001, one-way ANOVA).

Within-breath phasic tongue muscle activity in the presence of muscarine at the HMN was significantly lower compared to when either atropine or scopolamine were co-applied at the HMN (p = 0.027 and p < 0.001, respectively, post hoc Holm–Sidak tests), i.e., indicating statistically significant marked antagonism of muscarinic receptor inhibition at the HMN by atropine and scopolamine respectively (Fig. 15a).

In contrast, the relative efficacy of oxybutynin (i.e., 10 μM and 100 μM) and omadacycline (i.e., 10 μM, 100 μM, and 1 mM) in antagonising muscarinic receptor inhibition were reduced and not statistically different from activity in the presence of muscarine per se (each p ≥ 0.957, post hoc Holm–Sidak tests, Fig. 15a). In addition, comparisons across each of oxybutynin and omadacycline, respectively, did not identify any significant differences between doses within each group (each p ≥ 0.892, post hoc Holm–Sidak tests), and neither did comparison of oxybutynin to omadacycline at all doses (each p ≥ 0.834, post hoc Holm–Sidak tests). Overall, these data showed that there was comparable efficacy of oxybutynin and omadacycline at the HMN in the presence of muscarinic receptor stimulation, and that the antagonism was reduced (less efficacious) compared to atropine and scopolamine, the broad-spectrum muscarinic receptor subtype antagonists.

There was no difference between males and females with muscarine at the HMN targeting only muscarinic receptors (t_17_ = 0.193, p = 0.849, unpaired t-test). This lack of sex-difference with muscarine was unlike the larger motor inhibitory responses in males compared to females in the presence of eserine-induced increased endogenous acetylcholine at the HMN (which would modulate both muscarinic and nicotinic receptors). Given this lack of sex-specific response to muscarine compared to eserine (Figs. 15 vs. 8) further studies with muscarine in females in the subsequent protocols (*3.2a-d*) were not performed in line with local animal care guidelines to reduce unnecessary experiments and animal numbers.

#### Other respiratory parameters

As shown in Fig. 15b,c, there were no significant differences across protocols for respiratory rate or diaphragm amplitude, except for the one comparison indicated (see symbol ‘*’, p = 0.007, post hoc Holm–Sidak test after one-way ANOVA identified significant differences between groups: p = 0.006). Additionally, there was a significant difference in respiratory rate between male and female rats with muscarine at the HMN (lower rate in males, Fig. 15b, p = 0.018, post hoc Holm–Sidak test after significant one-way ANOVA, F_1,18_ = 6.79, p = 0.018).

## Discussion

The first major impetus for the present study was the common assumption and/or underlying rationale from previous human studies and their accompanying editorials that the antimuscarinic agents in OSA pharmacotherapy are exerting their effects, at least in some significant part, at the HMN^11–14,16,18–20,22,29^. This assumption and rationale is largely based on previous animal studies: scopolamine-induced muscarinic receptor antagonism at the HMN leads to marked increases in tongue motor tone in sleep, especially rapid eye movement (REM) sleep, and restoration of physiological patterns and levels of activity in REM sleep that are typical of non-REM sleep^10^. The second major impetus was that effectiveness in improving human OSA severity and converting obstructive apneas to hypopneas or resolving apneas varies with muscarinic receptor subtype affinities of the agents administered^11–14,16,17^. For example, clinical studies using a variety of agents with different antimuscarinic properties (such as biperiden hydrochloride, M1 or solifenacin succinate, M2 and M3) when combined with atomoxetine may themselves not change apnea hypopnea index but convert obstructive apneas to hypopneas and milder events^14^, unlike the more significant improvements with oxybutynin or scopolamine^11,13,15^. Overall, increased knowledge of cholinergic effects operating at the HMN, and their pharmacological manipulation, may translate to improvement or refinement of current pharmacological strategies and receptor subtype targets in future human studies and larger trials.

Potential sex-dependent differences in HMN responses to cholinergic modulation were unknown in previous human, rodent, or other such studies to our knowledge. It is known that there is a sex difference in human OSA prevalence, with increased incidence in males, and female incidence increasing post menopause^2,30,31^. Studies in rats have identified sex hormone effects on hypoglossal motor activity and the control of breathing^32–34^. Here we identify in rats that eserine-induced increased endogenous acetylcholine at the HMN in vivo elicits suppression of tongue motor activity, an effect that is sex dependent with lesser suppression in females versus males. Eserine was chosen given the physiological relevance of increasing endogenous acetylcholine to modulate all locally present nicotinic and muscarinic receptors and their subtypes. We identify that cholinergic-induced hypoglossal motor suppression was significantly attenuated by each of the selected FDA-approved muscarinic receptor antagonists at the HMN, including oxybutynin. Notably, oxybutynin was initially identified as a key component of effective OSA pharmacotherapy but with unknown effects at the HMN^11,15,16,22^. Notably also in the present study in rats, the residual tongue motor activity after atropine (broad spectrum muscarinic receptor antagonist), oxybutynin and omadacycline (M2 antagonist) at the HMN were statistically similar across agents in the presence of eserine-induced cholinergic inhibition via increased endogenous acetylcholine. This result suggests the capacity for similar efficacy at the HMN in modulating motor activity despite some differences in muscarinic subtype affinities and the sparing of nicotinic receptor-mediated excitation.

Despite the significant attenuation of cholinergic inhibition in the presence of each of these antimuscarinic agents at the HMN in the present study, the sex differences remained with atropine and oxybutynin (lesser cholinergic suppression in females) but not with omadacycline (similar cholinergic suppression in females and males). In contrast, the primary muscarinic receptor-mediated inhibition elicited by muscarine at the HMN was not sex dependent. The similar efficacy of oxybutynin and omadacycline also occurred in the presence of muscarine at the HMN across the range of doses tested. In the presence of muscarine at the HMN, scopolamine is capable of full blockade of cholinergic inhibition (Fig. 15) and capable of preventing the strong HMN inhibition that accompanies REM sleep in rats^10^. It is currently unknown if oxybutynin, or other agents with more specific M2 affinities such as omadacycline, is (are) similarly capable of preventing cholinergic inhibition of the HMN in REM sleep in animal studies. Omadacycline was only used here as a recently FDA-approved agent^23^ for mechanistic interrogation of M2 receptor targets^24^, although it is acknowledged that other agents could also have been selected. Such proof-of-concept may stimulate testing of agents with central activity and M2 specificity with a view to potential activation of pharyngeal motor activity in sleep in combination with noradrenergic reuptake inhibitors. However, it is recognized that both atomoxetine and oxybutynin, for example, can increase cardiovascular activity although minimal changes were observed in OSA patients: 2 beats per minute in heart rate and no change in blood pressure^11^. Such concerns will need to be followed clinically with cholinergic-noradrenergic pharmacotherapy for OSA and effects may vary, being more favourable with some combinations than others depending on receptor subtype-targeting profiles and length of treatment, with relatively short duration published studies in humans to date.

Cholinergic influences at the HMN include a combination of muscarinic and nicotinic receptor-mediated effects^25,35–40^. The *net* effects of cholinergic modulation of the HMN on motor output to the tongue musculature is complex, with muscarinic receptor-mediated inhibition dominating nicotinic receptor-mediated excitation in rodents in vivo^25^ and in vitro^26,27^. Initial immunohistochemical and in-situ hybridization studies indicated strong abundance of proteins or mRNAs for the M2 receptor on brainstem motoneurons with relatively little for M1, M3, M4 and M5^41^. Subsequent immunohistochemical studies focussing on the HMN indicate the presence of M1, M2 and M5 at the HMN in neonatal rats^42^. Muscarinic receptor mediated inhibition of motoneurons occurs via two major mechanisms: (i) pre-synaptic muscarinic inhibition via M2 receptors produces dis-facilitation of spinal^43^ and hypoglossal motoneurons^26,27^, and (ii) post-synaptic M2 receptor activation inhibits motoneurons via induction of inwardly rectifying potassium current^26,44,45^. Both such effects likely explain, at least in great part, previous^10^ and current effects of cholinergic agents at the HMN in rats in vivo. M2 receptors are the most highly expressed at the rodent HMN and the major pre-motor inputs to the HMN^46^. Notably, pre-synaptic M2 receptors inhibit excitatory glutamate inputs to hypoglossal motoneurons^26,27^ that mediate transmission of excitatory respiratory drive^47,48^. The dominant muscarinic receptor-mediated inhibition operating at the adult rat HMN appears to overcome any muscarinic (non-M2) excitatory effects as identified for neonatal hypoglossal motoneurons^26,39^.

Overall, the present findings and positive responses to antimuscarinic agents in OSA pharmacotherapy in patient populations^11–15^ should stimulate future work to combine a full molecular identification of each of the muscarinic receptor subtypes with their pre and/or post-synaptic locations at the HMN, with identified effects on known inputs (e.g., excitatory glutamatergic inputs mediating the respiratory drive signal) and full characterization of the pharmacological profiles of the applied antimuscarinic agents. Such work is needed to further define and refine targeted pharmacotherapeutic approaches to optimize modulation of HMN output to the tongue musculature for OSA pharmacotherapy^14,16,46^. It is important to note, however, that some component of the positive responses to antimuscarinic drugs used clinically could be due to effects at the HMN and/or elsewhere, the latter including upstream effects altering other factors that improve sleep apnea (e.g., arousal threshold or loop gain)^14,16,46^. Those upstream effects could involve muscarinic receptor subtypes targeted by the antimuscarinic agents used clinically but those subtypes may not be present at the HMN. The contribution of the present study is to characterize effects of the selected agents at the HMN.

Increased endogenous acetylcholine at the HMN in the presence of muscarinic receptor antagonists would leave nicotinic excitation intact. Differences in nicotinic receptor function between males and females may therefore contribute to the lesser motor suppression observed after co-application of atropine or oxybutynin in the presence of eserine-induced cholinergic inhibition via increased endogenous acetylcholine. By itself, however, such a difference would not explain the lack of sex difference with the M2 antagonist omadacycline also in the presence of eserine-induced endogenous acetylcholine. Sex differences in excitatory muscarinic (non-M2) receptors may, therefore, also contribute.

Administration of antimuscarinic agents *alone* to OSA patients does not improve OSA severity, and likewise with noradrenergic reuptake inhibitors alone: both agents need to be applied together for successful OSA pharmacotherapy^11^. This requirement for combination therapy may be based on physiological precedent and known mechanisms. Studies across natural sleep–wake states in rats identify an endogenous noradrenergic (α_1_ receptor-mediated) drive to the HMN that contributes to tongue motor activity in wakefulness, with this contribution withdrawn in non-REM sleep and further reduced to a minimum in REM sleep^6,9^. In contrast, the contribution of muscarinic receptor-mediated inhibition of hypoglossal motor output to the tongue musculature in rats is strongest in REM sleep but also present, to a lesser degree, in non-REM sleep^10^. As such, although the relative net contributions of noradrenergic excitation and muscarinic inhibition to net hypoglossal motor output may vary inversely to each other in states of non-REM and REM sleep, modulation of both would be expected to result in higher net activity than either alone across the sleeping period and, if applicable from rats to humans, explain why the agents need to be applied together for successful OSA pharmacotherapy^11^.

Notably, three randomized, double-blind, placebo-controlled human studies have shown that OSA severity can be reduced by administration of acetylcholinesterase inhibitors^49–51^. Nevertheless, the improvements were variable, likely reflecting the multi-factorial nature of OSA and the varying contributions of the four major endotypes operating within and between patients: pharyngeal anatomy, pharyngeal dilator muscle activity, arousal threshold and loop gain^52–55^. Given the consistent findings from the present study (Figs. 2, 3 and 8), and previous studies in rats both under general anesthesia^25^ and across sleep–wake states^56^, that acetylcholinesterase inhibition at the HMN suppresses tongue motor activity, it would be unlikely that any beneficial effects of acetylcholinesterase inhibitors in OSA patients would be due to effects at the HMN. Rather, it would be expected that acetylcholinesterase inhibition would be harmful, or of minimal benefit, in those OSA patients in whom the dominant endotype that contributes to their pathophysiology is pharyngeal dilator muscle tone and responsiveness. As such any beneficial effects of acetylcholinesterase inhibition in OSA patients likely originate from effects on other endotypes and not effects at the HMN.

The present study was performed in anaesthetized rats to allow measurements of tongue motor activity during controlled delivery of a variety of cholinergic agents into the HMN across multiple related protocols, without the influence of unpredictable changes in ongoing behavioural state and spontaneous motor behaviours that typify the conscious preparation. The levels of within-breath phasic tongue motor activity are also robust and of larger magnitude with isoflurane anaesthesia than without anaesthesia^48,57,58^, thereby making any motor suppression-effects easier to detect, quantify and compare across the 13 protocols during comparable time-frames, conditions and relative stability. Time controls showed that tongue (and diaphragm) motor activity was stable throughout the experiments (Figs. 2, 7, 8, 9, 15) indicating that the changes observed with the cholinergic interventions were due to the interventions per se and not the effects of time. Changes in respiratory rate were sometimes observed during the experiments but these also occurred during the ACSF time controls (Figs. 7, 8 and 15) and as such appeared due to the general effects of time during anesthesia and not the drug interventions per se.

Respiratory-related tongue motor activity was present across experiments in all protocols in these anesthetized rats but tonic activity was not present, typical of other studies^25,28,48,58^. Muscarinic receptor antagonism at the HMN in sleeping rats leads to an increase in within-breath phasic (respiratory-related) tongue muscle activity in non-REM with larger effects in REM sleep, as well as increased tonic motor activity in phasic REM sleep^10^. Nicotinic receptor agonism at the HMN in rats via microperfusion of dimethyl-4-phenylpiperazinium iodide (DMPP) increases tonic as well as respiratory-related tongue motor activity^25^ but tonic activity was never elicited by any intervention in the present study (e.g., Fig. 2). This finding is in agreement with the previous demonstration in rats that application of (i) carbachol (mixed nicotinic and muscarinic receptor agonist), (ii) acetylcholine, or (iii) eserine to the HMN do not increase tonic activity but instead suppress respiratory-related activity due to the dominance of muscarinic receptor-mediated inhibition^25^. The lack of an effect on tongue motor activity of the muscarinic receptor antagonists applied alone to the HMN compared to the activity recorded during the baseline ACSF controls (Figs. 2, 4, 5 and 6) support the lack of a tonically active endogenous cholinergic inhibition at the HMN operating under general anesthesia^25^.

The source and operation of the cholinergic respiratory and sleep-related modulation of the HMN in vivo is currently unknown. There are two main groups of premotor cholinergic inputs to the HMN: (i) the intermediate reticular nucleus (IRt) in the lateral medulla, and (ii) the pedunculopontine tegmental (PPT) and laterodorsal tegmental (LDT) nuclei in the pons^59–64^. The IRt is the critical relay for the transmission of respiratory drive to the HMN and hypoglossal nerve *in-vitro*^65–68^. Some hypoglossal pre-motor IRt neurons are cholinergic^62^ and may be a source of HMN inhibition as they remain relatively active in REM sleep in rats^69^. Muscarinic inhibition also suppresses excitatory glutamatergic IRt inputs to the HMN in rodents^26,27^. PPT and LDT neurons are active in REM sleep and modulate REM sleep expression^70,71^ by facilitating stable transitions^72,73^. Some cholinergic PPT/LDT neurons innervate the HMN^63^ but their role in modulating tongue motor activity is currently unknown in vivo.

Together, the results of the present study identify differential pharmacological and sex-specific effects of select antimuscarinic agents at the HMN in rats in vivo. The results have translational relevance to understanding current and potential future directions for antimuscarinic OSA pharmacotherapy. Sex differences in HMN suppression with increased endogenous acetylcholine at the HMN but not muscarine may relate to the sparing of accompanying nicotinic excitation.

## Methods

Experiments were performed on a total of 256 Wistar rats (Charles River, Senneville, QC, Canada) of which data from 181 subjects were included for analysis (70.7% success rate). The inclusion criteria for the data from an individual animal to be included in the analysis required that each of the following be met: (i) a stable 30-min baseline control period following insertion of the microdialysis probe into the HMN, (ii) a positive motor activation response to microperfusion of 5-hydroxytryptamine (5-HT) into the HMN at the end of the experiment (i.e., a positive control), and (iii) positioning of the microdialysis probe membrane within the HMN as confirmed by histology. All procedures conformed to the recommendations of the Canadian Council on Animal Care, and the protocols were approved by the University of Toronto Animal Care Committee. The studies and their reporting followed the ARRIVE guidelines^74^. The inclusion criteria for a technically proficient experiment identified above were met for 121 males (312.1 ± 1.9 g, 270–380 g, mean ± standard error of the mean (SEM), range) and 60 females (260.0 ± 2.2 g, 220–295 g) yielding the population of 181 subjects studied across the 13 separate protocols.

### Animal preparation

Animals were first anesthetized in an induction chamber using 3.5% isoflurane for approximately five minutes followed by 2.5% for another 10 min. Following induction of general anesthesia as judged by the abolition of pedal withdrawal reflex to paw pinch, the animals were placed on a heating pad for the experiments for warming for the duration of the experiments (T/Pump Model #TP500, Gaymar Industries, Inc., Orchard Park, NY, USA). The rats breathed a 1:1 mixture of air and oxygen, and general anesthesia was maintained with isoflurane (male: 2.36 ± 0.04%, 1.4–4%; female: 2.26 ± 0.09%, 2–2.6%) delivered via a snout mask (Kopf model 923, Tujunga, CA, USA) for the remainder of the experiment.

To record diaphragm electromyogram (EMG) activity, bipolar stainless-steel electrodes (AS636, Cooner Wire, Chatsworth, CA, USA) were sutured onto the right side of the costal diaphragm via an abdominal approach. Two stainless-steel needle electrodes (Natus Medical, Middleton, WI, USA) were also inserted bilaterally into the tongue musculature via a per-oral approach to record EMG activity of the tongue musculature. The rats were positioned in a stereotaxic apparatus (Kopf model 940, Tujunga, CA, USA) using blunt ear bars and the skull leveled. Two electroencephalogram (EEG) electrodes attached to small screws were implanted over the frontal-parietal cortex to measure EEG activity.

### Manipulation of the HMN

#### Microdialysis perfusion

For local administration of pharmacological agents into the HMN, microdialysis probes (CX-I-12-1, Eicom, San Diego, CA, USA; 0.22 mm outer membrane diameter, 1 mm membrane length with a 50,000 Daltons molecular weight cut-off) were used. The probes were connected to fluorinated ethylene propylene (FEP) Teflon tubing (0.1 mm inside diameter, 1 m length) which in turn were connected to 1.0 mL syringes via a zero dead-space switch (Uniswitch, B.A.S. West Lafayette, IN). The syringes were mounted on a syringe pump (MD-1001, B.A.S. West Lafayette, IN, USA) that regulated the flow of the perfusion medium via a controller (MD-1020, B.A.S. West Lafayette, IN, USA).

#### Targeting the HMN

Prior to insertion into the brain, the microdialysis probes were initially perfused with distilled water for 10 min, and subsequently with ACSF at a flow rate of 2.1μL/min to document continuous flow and no blockage. The composition of the ACSF was 125 mM NaCl, 3 mM KCl, 2 mM CaCl_2_, 1 mM MgSO_4_, 25 mM NaHCO_3_, and 30 mM glucose with a pH value of 7.41 ± 0.0, 7.31–7.5^25^. The microdialysis probes were placed through a small hole drilled at the junction of interparietal and occipital bones at the following midline coordinates: 14.1 ± 0.02 mm (13.4–15.0 mm) posterior to bregma, and 9.51 ± 0.03 mm (8.2–10.6 mm) ventral to bregma. A brief burst of tongue muscle activity (lasting on average 2 min and 58 s ± 24.4 s; 40–900 s) was observed once the probe penetrated the HMN, with no effects observed on diaphragm activity or respiratory rate. This response is expected and typical, and is used as a preliminary confirmation of probe placement into the HMN prior to subsequent determination by histology after the experiment^75,76^.

Following insertion of the microdialysis probe, ACSF was microperfused into the HMN. The signals were monitored closely until at least a 30-min period of stable tongue and diaphragm EMG activities, respiratory rate and EEG activity were observed before any experimental interventions were performed. This period was designated as the baseline control condition for each experiment. The specific pharmacological agents of interest (see separate protocols below) were then microperfused into the HMN at a flow rate of 2.1 μL/min and a lag time of 5.4 min. The lag time was taken as the duration for the perfusion medium to reach the tip of the microdialysis probe following a switch between drugs and was calculated using the known flow rate and volume of the tubing.

#### Electrophysiological recordings

The electrophysiological signals were amplified and filtered (Super-Z head-stage amplifiers and BMA-400 amplifiers/filters, CWE Inc., Ardmore, PA, USA). The bandpass filters for the EEG and EMG signals were 1–100 Hz and 100–1000 Hz, respectively. The moving-time averaged signals of the tongue and diaphragm signals were also obtained with a time constant of 100 ms (MA-821/RSP Moving Averager, CWE Inc.). The electrocardiogram artifact was removed from the diaphragm EMG using an electronic blanker (Model SB-1, CWE Inc.). Using a CED 1401 data acquisition system, the raw analog signals were digitized at a sample rate of 1984 Hz and stored for analysis (CED 1401 and Spike 2 software, version 6.07, Cambridge Electronic Design Ltd., Cambridge, UK).

#### Protocols

Each protocol consisted of three 30-min intervention periods (i.e., 25 min for the experimental period plus an additional 5 min to allow for the lag time for drug delivery from the point of switching the perfusate) during which agents of interest were microperfused into the HMN. The first intervention in all protocols was the baseline control condition with microperfusion of ACSF into the HMN.

### Study 1: Cholinergic modulation of the HMN by eserine and effect of select muscarinic receptor antagonists

#### Protocol 1.1: Tongue motor inhibition by eserine

*Protocol 1.1* was performed to quantify the net inhibitory effects of increasing *endogenous* levels of acetylcholine on tongue motor activity by local microperfusion into the HMN of 100 μM eserine (acetylcholinesterase inhibitor, molecular weight (MW): 324.39 g/mol, catalogue number: 0622, Tocris)^25^. Experiments were performed in nine male rats (306.7 ± 5.48 g, 290–336 g) and 10 females (250.0 ± 4.1 g, 230–270 g). The sequence for this protocol consisted of: (i) ACSF 1; (ii) ACSF 2 (to be consistent with the timeline of *Protocol 1.2* where select muscarinic receptor antagonists are microperfused into the HMN); (iii) 100 μM eserine.

Notably, application of acetylcholinesterase inhibitors to the HMN to increase endogenous acetylcholine levels leads to muscarinic receptor-mediated inhibition of genioglossus activity that dominates nicotinic receptor-mediated excitation^25–27^.

#### Protocol 1.2: Modulation of eserine-induced cholinergic hypoglossal motor inhibition by select antimuscarinic agents at the HMN

*Protocols 1.2a-c* were performed to quantify the relative efficacy of three muscarinic receptor antagonists at the HMN in reducing tongue motor inhibition by eserine-induced increased acetylcholine.

#### Protocol 1.2a: Atropine

Experiments were performed in nine male rats (313.9 ± 4.0 g, 302–343 g) and 10 females (260.5 ± 6.3 g, 230–295 g). This protocol consisted of: (i) ACSF; (ii) 10 μM atropine, a muscarinic receptor antagonist (MW: 694.83 g/mol, catalogue number: A0257, Sigma) with high affinity for all subtypes^77^; (iii) 10 μM atropine co-applied with 100 μM eserine.

#### Protocol 1.2: Oxybutynin

Experiments were performed in nine male rats (311.1 ± 9.0 g, 280–360 g) and 10 females (268.0 ± 5.5 g, 240–290 g). This protocol consisted of (i) ACSF; (ii) 100 μM oxybutynin (MW: 393.95 g/mol, catalogue number: O2881, Sigma); (iii) 100 μM oxybutynin co-applied with 100 μM eserine. Oxybutynin is a muscarinic receptor antagonist with affinities across the range of muscarinic receptor subtypes^21^ and a component of effective OSA pharmacotherapy^11,15,16,22^.

#### Protocol 1.2c: Omadacycline

Experiments were performed in 13 male rats (307.4 ± 7.1 g, 280–380 g) and 13 females (251.9 ± 5.4 g, 220–280 g). This protocol consisted of: (i) ACSF; (ii) 100 μM omadacycline (muscarinic receptor antagonist, MW: 556.65 g/mol, product code: 24,932, Cedarlane); (iii) 100 μM omadacycline co-applied with 100 μM eserine. Omadacycline is a recently FDA-approved agent and a selective M2 receptor antagonist^23,24^.

### Study 2: Time controls

#### Protocol 2: Time controls with continued microperfusion of ACSF into the HMN

In *Protocol 2,* repeated switches to microperfusion of ACSF into the HMN (i.e., ‘sham interventions’) were performed over the same time course as the drug interventions in *Protocols 1.1* and *1.2a-c*. These experiments were performed to determine if there were any effects of time per se on respiratory motor activities independent of the drug interventions. Experiments were performed in 13 male rats (299.2 ± 5.5 g, 270–340 g) and six females (269.2 ± 4.6 g, 260–290 g). This protocol consisted of three repeated ACSF interventions at the HMN, i.e., ACSF 1, ACSF 2, and ACSF 3.

### Study 3: Cholinergic modulation of the HMN by muscarine and effect of select muscarinic receptor antagonists

#### Protocol 3.1: Tongue motor inhibition by muscarine

*Protocol 3.1* was performed to quantify the effect of muscarinic receptor stimulation on tongue motor activity by the local microperfusion into the HMN of muscarine (muscarinic receptor agonist, MW: 209.71 g/mol, catalogue number: M6532, Sigma)^25^. Experiments were performed in eight male rats (320.6 ± 4.1 g, 300–335 g) and 11 females (265.9 ± 2.8 g, 250–280 g). This protocol consisted of a sequential order of microperfusion into the HMN of: (i) ACSF 1 (i.e., baseline control condition); (ii) ACSF 2 (consistent with the timelines in other protocols where antagonists are also microperfused before the agonists); (iii) 100 μM muscarine.

#### Protocol 3.2: Modulation of muscarine-induced hypoglossal motor inhibition by select antimuscarinic agents at the HMN

*Protocols 3.2a-d* were performed to quantify the relative efficacy of four muscarinic receptor antagonists at the HMN in reducing muscarine-induced tongue motor inhibition. Given the lack of sex-specific responses to muscarine compared to eserine (see “Results”) the subsequent protocols with muscarine (*3.2a-d* below) were not performed in both sexes (males only), in line with local animal care guidelines to reduce unnecessary experiments and animal numbers.

#### Protocol 3.2a: Atropine

Experiments were performed in eight male rats (305.6 ± 8.2 g, 280–350 g). This protocol consisted of a sequential order of microperfusion into the HMN of: (i) ACSF; (ii) 10 μM atropine; (iii) 10 μM atropine co-applied with 100 μM muscarine.

#### Protocol 3.2b: Scopolamine

Experiments were performed in 10 male rats (308.0 ± 5.7 g, 290–350 g). The sequence for this protocol consisted of: (i) ACSF; (ii) 1 mM scopolamine (broad spectrum muscarinic receptor antagonist, MW: 384.27 g/mol, catalogue number: 1414, Tocris)^10^; (iii) 1 mM scopolamine co-applied with 100 μM muscarine.

#### Protocol 3.2c: Oxybutynin

For this protocol one set of experiments was performed with 10 μM oxybutynin in seven male rats (315.0 ± 1.9 g, 310–320 g), and another set with 100 μM oxybutynin in a separate group of seven male rats (312.6 ± 4.1 g, 290–320 g). This protocol consisted of (i) ACSF; (ii) 10 μM or 100 μM oxybutynin; (iii) 10 μM or 100 μM oxybutynin co-applied with 100 μM muscarine.

#### Protocol 3.2d: Omadacycline

Three sets of experiments were performed with (i) 10 μM omadacycline in 10 male rats (319.0 ± 9.4 g, 290–370 g), (ii) 100 μM omadacycline in 10 male rats (333.5 ± 4.8 g, 320–370 g), and (iii) 1 mM omadacycline in eight male rats (311.25 ± 5.41 g, 295–340 g). This protocol consisted of (i) ACSF; (ii) 10 μM or 100 μM or 1 mM omadacycline; (iii) 10 μM or 100 μM or 1 mM omadacycline co-applied with 100 μM muscarine.

### Positive controls at the end of all protocols

At the end of each experiment in each protocol, 10 mM 5-HT (MW: 405.43 g/mol, catalogue number: H7752, Sigma) was microperfused into the HMN as a positive control to confirm the responsivity of the HMN and its ability to respond to the pharmacological interventions^78^. A positive response was defined as (at least) a doubling of the tongue EMG signal. Tongue EMG response to 5-HT occurred within 14 min and 45 s ± 31 s of switching to 5-HT microperfusion medium, consistent with previous studies^78^.

### Histology

At the end of the experiments, the rats were overdosed with 5% isoflurane for 20 min. The heart was then perfused with 30 mL of 0.9% saline and 30 mL of 10% formalin. The brainstem was removed and fixed in 10% formalin for a minimum of 24 h at room temperature for subsequent histological processing. Three days prior to tissue sectioning, the brainstem was transferred into a 30% sucrose solution for cryoprotection and stored at − 4 °C. Sectioning of the brainstem was performed in − 20 °C at 50 μm thickness using a cryostat (Leica, CM 1850, Wetzlar, Germany). The histological sections were then mounted on plus microscope slides (VWR, PA, USA), sealed with Cytoseal 280 (Thermo Scientific, Waltham, MA), and stained with neutral red dye (neutral red powder, 1 M CH_3_COOH, 1 M NACH_3_COO, 50% ethanol). A bright field microscope attached to a charge-coupled device (CCD) camera (Infinity 1 and BX-41, Olympus, Center Valley, PA, USA) was used to capture images using the Infinity Capture software (Lumenera, Ottawa, ON, Canada). The captured images of stained sections were cross referenced to a standard stereotaxic atlas of the rat brain^79^ to identify the anatomical placement of the microdialysis probes.

### Data analysis

Signals recorded during the last minute of each intervention at the HMN were used for data analysis^25,80^. The moving-time averaged tongue and diaphragm EMG signals were analyzed breath-by-breath over consecutive five-second time bins. Electrical zero, recorded as the voltage measured with the amplifier inputs grounded, was subtracted from the EMG signals. The analysis of tongue muscle EMG was time-locked to breathing (as defined by the peaks and troughs of the diaphragm signal) for each breath, quantified in volts, tabulated in a spreadsheet and matched to the intervention at the HMN^25,80^. The tongue EMG signals were quantified as tonic activity (minimal activity during expiration) and within-breath phasic activity (peak inspiratory activity minus tonic activity). Mean diaphragm EMG amplitudes (i.e., phasic respiratory diaphragm activity) and respiratory rates were also calculated^25,80^.

### Statistical analyses

Analyses were performed using *Sigmaplot* (version 14, Systat Software Inc., San Jose, CA, USA). To test for differences between multiple interventions in each animal, a one-way analysis of variance with repeated measures (ANOVA-RM) was performed followed by Dunnett’s test for post hoc comparisons. If data were not normally distributed, a one-way ANOVA on ranks was performed followed by Dunnett’s test for post hoc comparisons. For comparisons between protocols a one-way ANOVA was performed on normalized data. Two-way ANOVAs were used to identify sex effects on the responses to the interventions, with Holm–Sidak tests for pairwise post hoc comparisons. Unpaired t-tests were also performed as appropriate where indicated. For all tests, differences were considered significant if the null hypothesis was rejected at p ≤ 0.05.

## Data Availability

Generated raw data and/or analyzed data from the current study are available from the corresponding author on reasonable request.
